# Draft Genome Sequence of Methicillin-Resistant Clinical *Staphylococcus aureus* Isolate 51S (Sequence Type 291)

**DOI:** 10.1128/genomeA.01050-15

**Published:** 2015-09-24

**Authors:** Bernard S. Marasa, Saeed Khan, Saira Iram, Kidon Sung, Joshua Xu

**Affiliations:** a Division of Microbiology Assessment, Office of Process and Facilities, Center for Drug Evaluation and Research (CDER), U.S. Food and Drug Administration, Silver Spring, Maryland, USA; b Department of Microbiology, Quaid-i-Azam University, Islamabad, Pakistan; c Division of Microbiology, National Center for Toxicological Research (NCTR), U.S. Food and Drug Administration, Jefferson, Arkansas, USA; d Division of Bioinformatics and Biostatistics, NCTR, U.S. Food and Drug Administration, Jefferson, Arkansas, USA

## Abstract

We report the draft genome sequence of a methicillin-resistant clinical *Staphylococcus aureus* isolate with a novel *spa* type and sequence type (ST291), isolated from a renal failure patient in Rawalpindi, Pakistan.

## GENOME ANNOUNCEMENT

*Staphylococcus aureus* is one of the leading human pathogens, equipped with antimicrobial resistance and virulence genes (1), that causes a broad variety of infections, ranging from minor infections of the skin to life-threatening conditions, such as endocarditis, pneumonia, toxic shock syndrome, and bloodstream infections (2, 3). *S. aureus* isolate 51S was a methicillin-resistant nasal isolate from a chronic renal failure patient in Rawalpindi, Pakistan. To study the evolution of methicillin-resistant clinical *Staphylococcus aureus* (MRSA) clones that have emerged since the early 1960s, and their subsequent worldwide dissemination, it is important to have a library of representative genome sequences of these pathogenic bacteria as references. Genome sequences of MRSA and other pathogenic microorganisms will not only enhance rapid diagnosis but also enable researchers to detect pathogen evolution and develop strategies for better therapeutic interventions.

Antimicrobial susceptibility profiling indicated that *S. aureus* 51S is a multidrug-resistant clinical isolate that is resistant to methicillin, ciprofloxacin, neomycin, oxacillin, ampicillin, tetracycline, and penicillin but susceptible to erythromycin, gentamicin, and kanamycin (4). This *S. aureus* isolate lacks any detectable plasmids but was positive for the staphylococcal cassette chromosome *mec* type (SCC *mec* III and V) genes. It harbored various toxin genes (*eta*, *sea*, *seb*, *sec*, *hla*, *hlb*, *hld*, *hlg*, *pvl*, *lukS, lukF, lukM, lukE-D*, *sed*, *see*, *seg*, *seh*, and *tst*), adhesin genes (*ebpS*, *bbp*, *cna*, *clfA*, *clfB*, *fnbA*, *fnbB*, and *map-eap*), and virulence genes (*chp*, *ica*, *cfb*, and *v8*). Multilocus sequence typing (MLST) using seven gene loci (i.e., *arcC*, *aroE*, *glpF*, *gmk*, *pta*, *tpi*, and *yqiL*), as described in Enright et al. (5), indicated an ST 291 profile (3-37-19-2-20-26-32). The new *spa* repeat sequence (B1:Q1:B1:B1:M1:r34:r24:r34:r34:r17) was assigned using http://spatyper.fortinbras.us/.

The genomic DNA of *S. aureus* 51S was extracted using a Master Pure Gram-positive DNA purification kit (Epicentre Biotechnologies, Madison, WI, USA). The library was prepared using a TruSeq DNA library preparation kit (Illumina, San Diego, CA, USA) while a TruSeq paired-end cluster kit was used for the DNA preparation and cluster generation. Sequencing was carried out on an Illumina HiSeq 2500 system. CLC genomics workbench 6.5.1 (CLC Bio, Cambridge, MA, USA) was used for the *de novo* assembly of high-quality 100 bp paired-end reads and further genome annotation was performed using the annotation method GeneMarkS+ in the NCBI Prokaryotic Genome Annotation Pipeline (http://www.ncbi.nlm.nih.gov/genome/annotation_prok/).

The draft genome sequence of *S. aureus* 51S was 2,708,625 bp in length, distributed in 62 contigs (average coverage, 270.0×) with 2,739 genes, 2 rRNAs (16S, 23S), 27 tRNAs, 1 noncoding RNA (ncRNA), 93 pseudogenes, and 41 frame-shifted genes.

### Nucleotide sequence accession numbers.

This whole-genome shotgun project has been deposited at DDBJ/EMBL/GenBank under the accession number JSAJ00000000. The version described in this paper is version JSAJ00000000.1.
